# NEO adjuvant chemotherapy in breast cancer: What have we learned so far?

**DOI:** 10.4103/0971-5851.68846

**Published:** 2010

**Authors:** Nirmal V Raut, Nilesh Chordiya

**Affiliations:** 1 Department of Medical Oncology, Terna Sahyadri Hospital, Nerul, Navi Mumbai, India; 2 Department of Surgical Oncology, Terna Sahyadri Hospital, Nerul, Navi Mumbai, India

**Keywords:** *Breast cancer*, *neoadjuvant chemotherapy*, *review*

## Abstract

Neoadjuvant chemotherapy (NACT) in breast cancer has undergone continuous evolution over the last few decades to establish its role in the combined modality management of these tumors. The process of evolution is still far from over. Many questions are still lurking in the minds of oncologists treating breast cancer. This review analyzes the evidence from metaanlyses, major multiinstitutional prospective trials, retrospective institutional series and systematic reviews in breast cancer to determine the current standards and controversies in NACT. The most effective drugs, their advantages, issues and controversies in delivery as well as the criteria for response are reviewed. A summary of evidence-based consensus is presented and unresolved aspects are discussed.

## THE BIRTH OF NEOADJUVANT CHEMOTHERAPY (NACT): FROM HALSTEAD TO FISHER

The changing trends in management of locally advanced breast cancer actually reflect the paradigm shift in the understanding of the biology of the disease.

The Halsteadian concept of breast cancer, to begin with, as a localized disease prevailed at the end of the nineteenth century, the scene being dominated by the surgeons and the different radical surgical approaches with a hope of increasing survival.[1,2] However, contrary to their expectations, the 5-year overall survival continued to be 15–20%. A retrospective analysis of multiple case series concluded that the probability of cure was inversely proportional to initial stage of malignancy (i.e., T and N) without being influenced by the extent of radicality of thesurgery.[3–7] Studying the patterns of FAILURE lead to a better understanding of the biology of the disease and thus a multimodal approach came into vogue.

Preclinical studies being performed at the same time led to the recognition that metastatic deposits are established in patients months or years before diagnosis.[8,9]

The Fischer’s hypothesis that the disease was systemic from the very beginning ignited a holy grail search of cytotoxic agents. In various animal models, they demonstrated that removal of the primary tumor resulted in an increase in the labeling index in residual tumor cells and an increase in circulating growth-stimulating factors.[10] Administration of NACT and endocrine treatment to these animals impaired the increase in cell growth observed in residual tumor cells in untreated animals.

Introduced in the early 1970s as part of an integrated therapeutic approach to treat inoperable locally advanced breast cancer, primary, anterior, induction or NACT resulted in high responses and sufficient down-staging to allow mastectomy in some patients. The small number of pathological complete responders, which was contrary to expectations, is now the prime focus of NACT trials.

Gradually, the idea of preoperative chemotherapy was extended to include patients with large but operable early-stage breast cancer. This approach allows the tumor to be used as a measure of treatment response *in vivo*. More recently, the possibility has opened up for NACT to provide information on the use of clinical, pathological and molecular endpoints, which can be used as surrogate markers to predict the long-term outcome in the adjuvant setting.

Perhaps the most dramatic conceptual change in the approach to breast cancer treatment is the realization that breast cancer is a conglomerate of several molecularly defined syndromes, with distinct prognoses, clinical courses and sensitivity profiles to existing therapeutics. The anatomical accessibility of the breast provides the potential for serial biopsies to investigate molecular changes during treatment.

## ADVANTAGES AND DISADVANTAGES OF NACT

Theoretically, they can be summed up as follows:[11]

**Table:** 

Advantages	Disadvantages
Reduction in tumor volume	Clinical/radiological staging imprecise
Tumor down-staging	Overtreatment of small favorable tumors
*In vivo* assessment of tumor response	Extent of surgery not confirmed
Less-extensive surgical resection	Loss of prognostic significance of axillary nodal status
Postsurgical growth spurt abrogated	Unknown relevance of surgical margins
Earlier introduction of a systemic therapy	Large number of drugresistant cells present
Response to chemotherapy serves as a marker for long-term outcome	Delays effective local therapy
Multiple sequential sampling of primary tumor allows evaluation of biologic changes during chemotherapy	Response of primary tumor may not correlate with response of micrometastases

Let us review the literature for searching what level of evidence we have for these.

## DOES NACT IMPROVE OVERALL SURVIVAL?

Mieog *et al*. conducted a systematic review[12] including 10 studies with 4,620 randomized women and 1,139 estimated deaths [Table 1]. The authors concluded that there was no survival difference between NACT and adjuvant chemotherapy [HR 0 • 98 (95% confidence interval {c.i.} 0 • 87—1 • 09)].

**Table 1:** Impact of NACT on overall survival

Study	Overall survival rate		Weight	Hazard ratio
	Neoadjuvant	Adjuvant		
Neoadjuvant				
Danforth[13]	3 of 26	6 of 27	0.37	0.18
Broet[14]	55 of 200	60 of 190	9.55	0.79
Mauriac[15]	48 of 134	51 of 139	7.64	0.99
Woolmark[16]	221 of 742	218 of 751	40.20	1.02
Gianni[17]	32 of 451	30 of 451	5.39	1.06
Van der Hage[18]	111 of 350	104 of 348	18.57	1.09
Subtotal	470 of 1,903	469 of 1,905	31.73	1.00
Test of heterogeneity	X^2^=5.16;			
	*P*=0.40			
Test for overall effect	Z=0.06;			
	*P*=0.95			
Sandwich				
Cleator[19]	43 of 144	53 of 142	12.36	0.81
Semiglazov[20]	20 of 137	30 of 134	2.61	0.88
Gazet[21]	27 of 100	21 of 110	2.05	1.21
Enomoto[22]	3 of 20	3 of 25	0.45	1.61
Subtotal	93 of 401	107 of 411	18.47	0.89
Test of heterogeneity	X^2^=1.52;			
	*P*=0.68			
Test for overall effect	Z=0.87;			
	*P*=0.39			
Total	563 of 2,304	576 of 2,316	100	0.98
Test of heterogeneity	X^2^=7.26;			
	*P*=0.61			
Test for overall effect	Z=0.43;			
	*P*=0.67			

## TIME TO LOCOREGIONAL RECURRENCE

Eleven studies [Table 2] reported time to locoregional recurrence data on 5,041 randomized women and 570 estimated recurrences. There was a significant difference in favor of adjuvant chemotherapy Table 1. However, in three studies, more than one-third of the patients received exclusive radiotherapy and no surgery after complete tumor regression.[13,17,18] Because of inadequate locoregional treatment after excluding these three studies, the remaining eight studies demonstrated no difference in the locoregional recurrence rate between the neoadjuvant and adjuvant groups [HR 1 • 12 (95% c.i. 0 • 92—1 • 37)].

**Table 2:** Impact of NACT on locoregional recurrence

Study	Overall survival rate		Weight	Hazard ratio
	Neoadjuvant	Adjuvant		
Ostapenko[23]	1 of 50	3 of 50	0.72	0.38
Gianni[17]	8 of 438	22 of 875	5.43	0.75
Enomoto[22]	2 of 20	3 of 25	0.90	0.93
Woolmark[16]	108 of 742	96 of 751	36.90	1.15
Van der Hage[18]	49 of 350	44 of 348	16.77	1.16
Gazet[21]	24 of 100	104 of 348	18.57	1.09
20 of 110	5.19	1.21	31.73	1.00
Cleator[19]	13 of 44	9 of 142	4.01	1.50
Danforth[13]	3 of 26	2 of 27	0.90	1.58
Subtotal	208 of 1,870	199 of 2,328	70.82	1.12
Test for heterogeneity	χ^2^=3.22, 7 d.f;			0.88
	*P*=0.86			
Test for overall effect	Z=1.15;			
	*P*=0.25			
Inadequate local treatment				
Broet[14]	17 of 95	17 of 86	6.15	0.90
Broet[14]	49 of 200	37 of 190	15.25	1.31
Mauriac[15]	31 of 134	12 of 138	7.78	2.57
Subtotal	97 of 429	66 of 414	29.18	1.45
Test for heterogeneity	χ^2^=5.67, 2 d.f;			
	*P*=0.006			
Test for overall effect	Z=2.36;			
	*P*=0.02			
Total	305 of 2,299	265 of 2,742	100	1.21
Test for heterogeneity	χ^2^=10.76, 10 d.f;			
	*P*=0.38			
Test for overall effect	Z=2.24;			
	*P*=0.03			

## RATE OF LOCAL TREATMENT IN THE NACT AND ADJUVANT CHEMOTHERAPY ARM

There was a statistically significant decrease in the mastectomy rate [Table 3] in favor of NACT [RR 0.71 (95% c.i. 0.67—0.75)], representing a risk difference of 16.6% (95% c.i. 15.1—18.1) (NNT 6). Of the 1,549 assessable women, 397 (25.6% [95% c.i. 23.5—27.8)] had their surgical treatment down-staged. In 66 women, [4.3% (95% c.i. 3.3—5.3)], tumor progression necessitated more radical surgery than originally planned.

**Table 3:** Metaanalysis of neoadjuvant chemotherapy

Study	Overall survival rate		Weight	Hazard ratio
	Neoadjuvant	Adjuvant		
Cleator[19]	16 of 149	31 of 144	2.39	0.50
Broet[14]	22 of 95	31 of 96	2.47	0.64
Woolmark[16]	239 of 743	302 of 752	22.77	0.80
Van der Hage[18]	203 of 323	262 of 341	19.33	0.82
Jakesz[24]	71 of 214	85 of 209	6.52	0.82
Danforth[13]	15 of 26	16 of 27	1.19	0.97
Broet	73 of 200	66 of 190	5.13	1.05
Gazet[21]	11 of 100	9 of 110	0.65	1.34
Subtotal	470 of 1,903	469 of 1,905	60.46	0.82
Test of heterogeneity	χ^2^=9.43; 7 d.f.; *P*=0.22	199 of 2,328	70.82	1.12
Test for overall effect	Z=5.10;			0.88
	*P*<0.001			
Gianni[17]	154 of 438	579 of 875	29.30	0.53
Mauriac[15]	74 of 134	136 of 136	10.24	0.55
Subtotal	228 of 572	715 of 1,011	39.54	0.54
Test of heterogeneity	χ^2^=0.16, 1 d.f.; *P*=0.69	37 of 190	15.25	1.31
Test for overall effect	Z=11.32;	12 of 138	7.78	2.57
	*P*<0.001			
Total	878 of 2,422	1,517 of 2,870	100	0.71
Test of heterogeneity	χ^2^=53.66, 9 d.f.; *P*<0.001			
Test for overall effect	Z=10.92;			
	*P*<0.001			
Total	305 of 2,299	265 of 2,742	100	1.21
Test for heterogeneity	χ^2^=10.76, 10 d.f; *P*=0.38			
Test for overall effect	Z=2.24;			
	*P*=0.03			

## RATE OF RESPONSE TO NACT

Here, we refer to two metaaanlysis performed by Davide Mauri[25] and Fredirica Cuppone.[26] The rates of complete clinical response were statistically significantly heterogeneous (ranging from 7% to 65%; *P* for heterogeneity of <0.001) across the studies [Table 4]. When both complete and partial clinical responses were considered, the difference between the extremes was smaller, but the rates were still statistically significantly heterogeneous (ranging from 45% to 83%; *P* for heterogeneity of <0.001).

**Table 4:** NACT and response rates

Study	Complete clinical response (%)	Partial clinical response (%)	Pathological response (%)
Avril, Mauriac[15]	33	30	unknown
Semiglazov[20]	12	57	29
Scholl[27]	13	32	unknown
Scholl[28]	24	42	unknown
Broet[14]			
Makris[29]	22	61	7
Woolmark[16]	36	43	13
Gazet[21]	25	26	unknown
Van der hage[18]	7	42	4
Danforth[13]	65	12	20

Thus, the conclusion from both these metaanalyses is that overall survival or disease-free survival (DFS) is not influenced by the timing of chemotherapy (before or after surgery) but is more likely to be influenced by the chemosensitivity of the primary lesion. The only benefit that neoadjuvant systemic therapy offers is the feasibility of breast conservation not at the cost of local recurrence, as thought earlier.

However, the recent update of the pioneering NSABP-18 study by Rastogi *et al*,[30] shows trends in favor of preoperative chemotherapy for DFS and OS in women less than 50 years old (hazard ratio 0.85, *P* 0.09 for DFS; HR 0.81, *P* 0.06 for OS).

## WHAT IS THE BEST CHEMOTHERAPEUTIC REGIMEN FOR NACT

The introduction of combination of multiple drugs was influenced from the Goldie Coldman hypothesis, according to which the risk of resistant tumor cells can be minimized by initiating a combination of non-cross-resistant drugs. In various nonrandomized and randomized trials employing primary chemotherapy, the most commonly used regimens were CMF/FAC/AC (C=Cyclophosphamide, A=Adraiamycin, F=5FU, M=Methotrexeate). Comparative trials in metastatic and adjuvant settings showed that the efficacy of anthracycline-containing regimens were highest in terms of response rates, DFS and OS.[31–33] The same was extrapolated in the neoadjuvant setting.

## ROLE OF TAXANES AS NACT

Federica Cuppone *et al*,[26] conducted a literature-based metaanalysis of randomized clinical trials (RCTs) to “weigh” how much taxanes add to anthracyclines as primary treatment over standard chemotherapy [Table 5]. Data from seven RCTs (2,455 patients) showed that the rate of Breast Conserving Surgery (BCS) was significantly higher for patients receiving taxanes, with an absolute difference of 3.4% (*P*=0.012), which translates into 29 patients NNT, without significant heterogeneity. The rate of Pathological complete response (pCR) was higher for patients receiving taxanes, although this was not statistically significant.

**Table 5:** Addition of taxanes to anthracyclines in NACT

Study	Stage of disease	No. of patients	Arms	ORR	pCR (%)
Malamos[34]	Operable	30/30	FEC	50	0
			ED	81	28
Aberdeen[35],	II B and III	162/104	CVAP	64	15
Smith[36]			CVAP-D	85	31
Luprosi[37]	II and III	90/50	FEC	72	24
			ED	84	24
NSABP-27[38,39]	II	1605/1605	AC-D	85	14
			AC	91	25
Evans[40]	II and III	365/363	AC	78	12
			AT	88	8
Semiglazov[20]	III A and III B	103/103	FAC	73	10
			AT	84	25
Dieras[41]	II A, II B and III A	247/200	AC	66	10
			AT	83	16

## IS THERE ANY ROLE OF DOSE-DENSE NACT?

The study by Citron *et al*.[42] has shown significant survival benefit with dose-dense chemotherapy in the adjuvant setting Table 7. However, such data in the neoadjuvant setting are sparse and the results are controversial.

**Table 7:** Dose dense NACT

Study	No.	Arms of the study	pCR	Rates of BCT
AGO Untch *et al*[43]	1,069 pts	Adria 150 mg/ m2 q2wkly for 3#‐>paclitaxel 250 mg/m2 q2wkly for 3#	*P*=0.03	P=0.016
		Adria 90 mg/ m2+docetaxel 175 mg/m2 q3wkly for 4#		
GEPARDUO[44]	931 pts	Adria 50 mg/ m2+docetaxel 75 mg/ m2 q2wkly for 4#	14.3%	63.4%
		Aria 60 mg/m2 and cyclophosphamide 600 mg/m2 q3wkly for 4# → docetaxel	10.6%	58.1%

## CAN ANTHRACYCLINES BE AVOIDED?

Anthracyclines, one of the most effective groups of agents for the treatment of breast cancer, should only be discarded or replaced on the basis of convincing data and, thus far, evidence to do so is lacking.

1. The US Oncology (USON) 9735 trial[45] compared four cycles of AC (doxorubicin 60 mg/m^2^) with four cycles of docetaxel (75 mg/m^2^) plus the same dose of cyclophosphamide (DC).[18,19] After 5.5 years of follow-up, DFS was significantly superior in patients treated with DC and after 7 years of follow-up, OS was also significantly better in the DC arm (88% *vs*. 84%; hazard ratio=0.73; *P*=0.045)[46]).
2. The BCIRG 006 trial[47] compared a nonanthracycline-containing taxane-based regimen [docetaxel, trastuzumab and carboplatin (TCH)] with two anthracycline–taxane combinations in patients with HER2-positive early breast cancer, but the study was designed primarily to evaluate the addition of trastuzumab, and the nonanthracycline-containing and anthracyclines-containing regimens differed in other ways.[30–38] Data from an interim analysis indicate that DFS and OS were significantly better in both trastuzumab arms compared with AC followed by docetaxel. There was no significant difference in efficacy between the two trastuzumab-containing arms, but there were fewer cardiac events and secondary leukemias with TCH.

## SHOULD ANTHRACYCLINES AND TAXANES BE USED CONCURRENTLY OR SEQUENTIALLY?

According to the reported results, a significant benefit in pCRs in favor of taxanes appears to be restricted to a sequential strategy (all of which used docetaxel) [Tables 6 and 8]. A trend in favor of taxanes was observed in the overall population as well, but the contribution of the sequential strategy was more than evident.

**Table 6:** Results: Primary end points and sensitivity analysis (fixed effect model)

	Patients (total no of pt’s)	Relative risk (95% CI)	*P* value	Heterogeneity	Absolute difference (%)	Number need to treat
*pCR*						
Overall	2455	1.22	0.11	0.05	-	-
concomitant	746	1.04	0.77	0.06	-	-
Sequential	1709	1.73	0.013	0.65	2.4	41
*BCS*						
Overall	2425	1.11	0.012	0.43	3.4	29
Concomitant	716	1.22	0.027	0.78	5.3	19

**Table 8:** Should anthracyclines and taxanes be used concurrently or sequentially?

	Effect name	Citation	Year	N total	*P*-value
	Concomitant-pCR	Malamos[34]	1998	30	0.27
	Concomitant-pCR	Luprosi[37]	2000	50	1.0
	Concomitant-pCR	Semiglazov[20]	2002	103	0.006
	Concomitant-pCR	Dieras[41]	2004	200	0.828
	Concomitant-pCR	Evans[40]	2005	363	0.469
Fixed	Concomitant-pCR			746	0.774
Random	Concomitant-pCR			746	0.422
	Sequential-pCR	Heys[35]	2002	104	0.063
	Sequential-pCR	Bear[38]	2006	1,605	0.075
Fixed	Sequential-pCR			1,709	0.013
Random	Sequential-pCR			1,709	0.013
Fixed	Combined			2,455	0.108
Random	Combined			2,455	0.117

## IS THERE ANY ROLE OF A NON-CROSS-RESISTANT CHEMOTHERAPY?

The Aberdeen group enrolled 162 locally advanced breast cancer patients to four cycles of CVAP (cyclophosphamide/vincristine/doxorubicin/prednisone. Of these, 66% of the patients who had clinical response were further randomized to four cycles of the same CVAP or four cycles of 3-weekly Docetaxel. Surgery performed at the conclusion of eight cycles found that there were significantly higher pathological complete remission rates, which also translated into a statistically superior survival rate. Thus, the study demonstrated that both the responders and the nonresponders to the initial chemotherapy regimen benefited from change over to a taxane-based chemotherapy.[35,36]

The GePAR TRIO study[47] subjected 2,090 patients of previously untreated breast cancer to two cycles of TAC. Patients whose tumors did not respond were further randomized to four cycles of TAC chemotherapy or a combination of capecitabine–vinorelbine. There was no statistical difference in the sonographic response, pathological complete response and rates of breast conservation in both the arms, concluding that addition of other agents to the anthracycline–taxane regimen in a sequential manner had no significant effect.

## SHOULD ALL THE CYCLES OF CHEMOTHERAPY BE DELIVERED PREOPERATIVELY?

The National Surgical Adjuvant Breast and Bowel Project Protocol B-27 randomly assigned women (N_2,411) with operable primary breast cancer to receive either four cycles of preoperative AC followed by surgery (group I) or four cycles of AC followed by four cycles of docetaxel, followed by surgery (group II), or four cycles of AC followed by surgery and then four cycles of docetaxel (group III)[38,39] [Table 9].

**Table 9:** Should all the cycles of chemotherapy be delivered preoperatively?

	Preop AC alone	Taxanes combination	*P*-value
*c*CR	40%	63%	<0.001
*p*CR	13%	26%	<0.001
% of pts with negative nodes	50%	58%	<0.001

Although the initial report in 2003 showed an increase in the pathological response rate when a taxane was added preoperatively,[38] the recent update by Rastogi *et al*. showed no impact on the OS and DFS.[30]

## WHAT IS THE IDEAL NUMBER OF CYCLES OF CHEMOTHERAPY TO BE DELIVERED PREOPERATIVELY?

In the GePAR TRIO study,[37] the first phase included randomization of responders to two cycles of TAC (*n*=1,390) initially and then to either a further of four or six cycles of TAC. The authors found no difference in the rates of pCR (21% *vs*. 23.5%; *P*=0.27) or breast conservation (67.5% *vs*. 68.5%; *P*=0.68). However the toxicity in the arm that received eight cycles was significantly higher. Hence, we conclude that probably six cycles of an active regimen is sufficient in the neoadjuvant setting.

## WHAT IS THE ROLE OF TARGETED THERAPY IN THE NEOADJUVANT SETTING?

There are three randomized studies till date in the neoadjuvant setting evaluating the role of additional trastuzumab to standard therapy [Table 10]. The M. D. Anderson study was stopped prematurely (after 42 of a planned 165 patients) because the pCR rate with trastuzumab added to paclitaxel followed by 5-fluoruracil-epirubicin-cyclophosphamide (*P*→FEC) chemotherapy was astriking 65% *vs*. 25%) with chemotherapy alone.[48]

**Table 10:** Reported randomized phase III trials with neoadjuvant trastuzumab

Reference	Number of patients	Patient population	Design	HER_2_ assessment	pCR rate, No H	percentage with H	(95% c.i.) *p*-value
Buzdar *et al*., 2005,[48] 2007[49]	42	65% T_2_ 40% N0/57% N_1_	P → FEC vs. P+H → FEC+H	IHC 3+ or FISH+	26 (9–51)	65 (43–84)	NS
Gianni et al., 2007[50]	228	60% T_4_ 85% N+	AP → P → CMF vs. AP+H → P+H → CMF+H	IHC 3+ or FISH	23 (NR)	43 (NR)	0.002
Untch *et al*., 2008[52]	453	NA	EC → D or EC → DX or EC → D → X vs. EC → D+H or EC → DX+H or EC → D+H → X+H	NA	20 (NR)	41 (NR)	<0.001

C, cyclophosphamide; CI, confidence interval; D, docetaxel; E, epirubicin; F, 5-fluoruracil; FISH, fluorescence in situ hybridization; H, trastuzumab; HER2, human epidermal growth factor receptor 2; IHC, immunohistochemistry; M, methotrexate; N, nodal status; NA, not applicable; NR, not reported; NS, not significant; P, paclitaxel; pCR, pathologic complete response; T, tumor size; X, capecitabine

The larger NeOAdjuvant Herceptin (NOAH) trial reported similar findings with trastuzumab added to doxorubicin-paclitaxel followed by paclitaxel followed by cyclophosphamide-methotrexate-5-fluoruracil (AP→P→CMF) chemotherapy.[50] Both these studies administered anthracycline chemotherapy concurrently with trastuzumab and did not report a high rate of observed cardiac toxicity, contrary to the 16% rate of clinical grade 3/4 congestive heart failure observed in the pivotal first-line metastatic trial with concurrent trastuzumab and doxorubicin cyclophosphamide (AC).[51] The GeparQuattro study evaluating epirubicin, cyclophosphamide and docetaxel with or without capecitabine and/or trastuzumab before surgery reported a similar doubling in the observed pCR rate with the addition of trastuzumab. This study initiates trastuzumab after the completion of anthracycline therapy.

**Figure 1:**
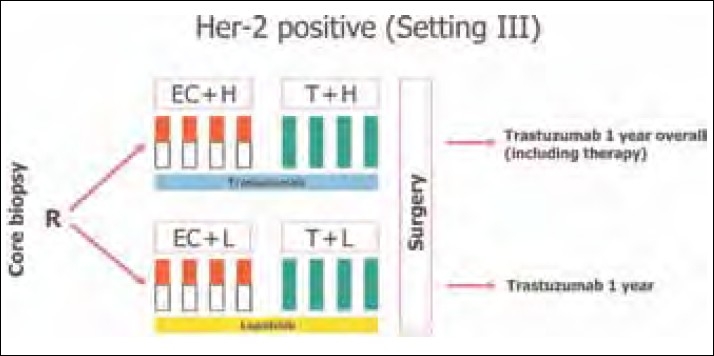
GeparQuinto study schema

**Figure 2:**
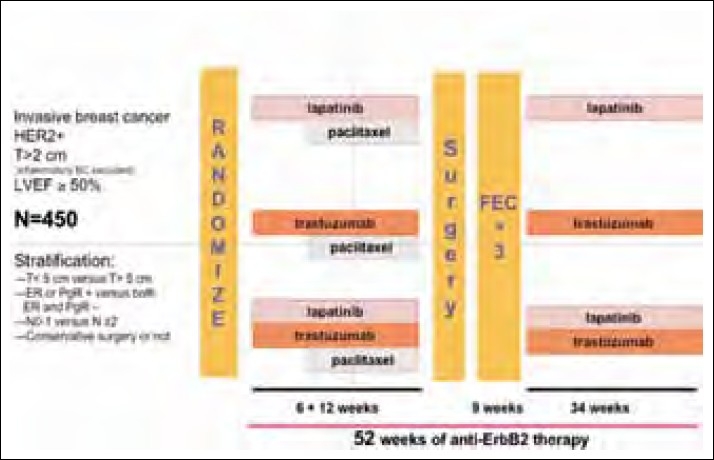
Neo ALLTO study schema

Two important ongoing neoadjuvant therapy trials are exploring the role of lapatinib in the neoadjuvant settings. Results are eagerly awaited. The schema of the study is shown in Figures 1 and 2.

GeparQuinto study (Ref Figure 1).

GeparQuinto study design for HER2-positive cohort. C, cyclophosphamide (600 mg/m^2^: day 1 q day 21 for four cycles); E, epirubicin (90 mg/m ^2^ : every 3 weeks for four cycles); H, trastuzumab (8 mg/kg: loading dose, 6 mg/kg: every 3 weeks); Her-2, human epidermal growth factor receptor 2; L, lapatinib (1,250 mg daily for 24 weeks: run-in phase cycles 1 and 5: 1,000 mg daily); R, randomization; T, docetaxel (100 mg/m^2^: every 3 weeks for four cycles).

## DOES ADDITION OF BEVACIZUMAB HELP?

Greil *et al*,[53] in a phase II study, studied the efficacy and safety of the combination of Bevacizumab, docetaxel and capecitabine for her2-negative breast cancer, and found a pCR of 22%.

## WHAT IS THE BEST WAY OF ASSESSMENT OF RESPONSE TO NEOADJUVANT THERAPY?

A study of 189 breast cancer patients undergoing NACT assessed tumor response to treatment with physical examination, mammography or ultrasound and compared these approaches with the gold standard, pathologic examination. The study found that false-positive rates ranged from 20% to 65% for all modalities; false-negative rates were 10-57%.[54] The GeparTrio trial[47] revealed a sonographic complete response in 50% of the cases examined, whereas a pathologic complete response was seen in only 5–6% of the patients.

Advantages of magnetic resonance imaging are that it provides evidence of response as early as 6 weeks of initiation of chemotherapy. Contrast enhancement is reduced even before actual reduction in the size of the tumor. However, the foible is that the accuracy varies with the degree of response to chemotherapy and with the chemotherapeutic agent, underestimating the response in well-responding tumors and taxane-based chemotherapy.[55–63] Several studies have shown the usefulness of Positron Emission Scan in the assessment of response.[64–69] A significant decline in the standardized uptake value occurs in responders early in the course of chemotherapy.

In a study of 22 patients, after an initial course of therapy, all responding (based on Standard Uptake Value changes) tumors were identified through a decrease in SUV of >55% below baseline (sensitivity, 100%; specificity, 85%).[68] Another study of 30 patients used PET at midtherapy assessments and reported a complete response, correlating with a 50—60% reduction from baseline SUV.[69]

However, outside a clinical trial, these approaches are not recommended for monitoring response of breast cancer to NACT.

The gold standard for assessing response to NACT for breast cancer is still pathologic evaluation.[3] Despite the proven predictive value of pCR in this context, there is no consensus on the measurement of this important endpoint. Three of the most commonly used criteria in the literature are those of Sataloff *et al*.,[7] Chevallier *et al*.[9] and Feldman *et al*.[4]

A study at M.D. Anderson[72] analyzed postmastectomy pathology specimens from 241 patients treated with neoadjuvant sequential paclitaxel followed by FAC regimen and 141 patients treated with a neoadjuvant FAC regimen. The investigators then calculated the residual cancer burden (RCB), which consisted of a continuous index combining primary tumor size and cellularity as well as number and size of nodal metastases. Using multivariate analysis, they showed that RCB correlated with prognosis, independent of factors such as age, pretreatment clinical stage, hormone receptor status, hormone therapy and pathologic response (hazard ratio: 2.5; 95% c.i. 1.7–3.69; *P* < 0.01). RCB was therefore proposed as a useful tool to estimate response to NACT in breast cancer because it provides a quantitative value of residual disease and has prognostic significance.

## NACT IN TRIPLE-NEGATIVE BREAST CANCER (TNBC)

TNBC is a heterogeneous, initially chemosensitive disease. Currently, there is no specific favored chemotherapy regimen for the treatment of TNBC. The use of taxane (paclitaxel or docetaxel) and anthracycline-based regimens, according to data for breast cancer patients in general, appear to provide higher pathological complete response rates. On the basis of the described similarities between sporadic triple-negative cancers and BRCA1-associated cancers, drugs with the ability to cause interstrand breaks, like platinum drugs, have been suggested to be used for the treatment of TNBC. This was supported by *in vitro* studies demonstrating the benefit of BRCA1-related tumors to these agents.[74] Because the availability of HER 2 testing is only of late, there are no studies for TNBC specifically. One study by Garber *et al*.[75] using preoperative single-agent cisplatin in T2/T3 TNBC reported a pCR of 23%.

A study by Carey *et al*.[76] evaluated responses to NACT in 107 patients with stages II and III breast cancer. Patients received neoadjuvant doxorubicin (60 mg/m^2^) plus cyclophosphamide (600 mg/m^2^) chemotherapy (AC) for four cycles, either alone or as the first component of a sequential AC-taxane neoadjuvant regimen. All patients received AC NACT at conventional doses for four cycles. Twenty-eight (26%) received AC on a dose-dense schedule (every 2 weeks), whereas the rest of the patients received AC on an every-3 weeks schedule. Most patients (80 of 107, 75%) received additional NACT following AC, which primarily involved either paclitaxel or docetaxel. PCR to chemotherapy (defined as postoperatively stage 0, no invasive cancer) was significantly better among basal-like subtype (27%), defined in this study as the immunohistochemical surrogates ER-, PR- and HER2/neu- and HER2/neu? /ER- (36%) subtypes vs. the combined luminal subtypes (7%; *P*=0.01). However, despite the initial chemosensitivity, patients with the basal-like and HER2/neu? /ER- subtypes had worse distant DFS (*P*=0.04) and OS (*P*=0.02) than those with the luminal subtypes This is known as the famous “Triple negative Paradox.” It has put to question all oncologists treating breast cancer who, until now, were using pCR as a surrogate for long-term survival.
